# Research on Physiological Characteristics and Differential Gene Expression of Rice Hybrids and Their Parents under Salt Stress at Seedling Stage

**DOI:** 10.3390/plants13050744

**Published:** 2024-03-06

**Authors:** Dan Zhang, Yuanyi Hu, Ruopeng Li, Li Tang, Lin Mo, Yinlin Pan, Bigang Mao, Ye Shao, Bingran Zhao, Dongyang Lei

**Affiliations:** 1 College of Agronomy, Hunan Agricultural University, Changsha 410128, China; mrsgrey@163.com; 2 State Key Laboratory of Hybrid Rice, Hunan Hybrid Rice Research Center, Changsha 410125, China; huyuanyi237@163.com (Y.H.); tangli@hhrrc.ac.cn (L.T.); mbg@hhrrc.ac.cn (B.M.); shaoye3183@163.com (Y.S.); 3 National Center of Technology Innovation for Salin-Alkali Tolerant Rice, Sanya 572000, China; 15531526788@163.com (R.L.); 18278032870@163.com (L.M.); panyinlin@foxmail.com (Y.P.); 4 School of Tropical Agricultture and Forestry, Hainan University, Haikou 570228, China

**Keywords:** rice, heterosis, salt tolerance, ROS, salt-related gene

## Abstract

Soil salinization is one of the most important abiotic stresses which can seriously affect the growth and development of rice, leading to the decrease in or even loss of a rice harvest. Increasing the rice yield of saline soil is a key issue for agricultural production. The utilization of heterosis could significantly increase crop biomass and yield, which might be an effective way to meet the demand for rice cultivation in saline soil. In this study, to elucidate the regulatory mechanisms of rice hybrids and their parents that respond to salt stress, we investigated the phenotypic characteristics, physiological and biochemical indexes, and expression level of salt-related genes at the seedling stage. In this study, two sets of materials, encapsulating the most significant differences between the rice hybrids and their parents, were screened using the salt damage index and a hybrid superiority analysis. Compared with their parents, the rice hybrids Guang-Ba-You-Hua-Zhan (BB1) and Y-Liang-You-900 (GD1) exhibited much better salt tolerance, including an increased fresh weight and higher survival rate, a better scavenging ability towards reactive oxygen species (ROS), better ionic homeostasis with lower content of Na^+^ in their Na^+^/K^+^ ratio, and a higher expression of salt-stress-responsive genes. These results indicated that rice hybrids developed complex regulatory mechanisms involving multiple pathways and genes to adapt to salt stress and provided a physiological basis for the utilization of heterosis for improving the yield of rice under salt stress.

## 1. Introduction

Salt stress is one of the most prevalent abiotic stresses. Soil salinization hinders the growth and development of various crops to varying degrees [1]. After exposure to salt stress, ionic toxicity and osmotic stress are induced in plants. This results in insufficient water and solute contents, the excessive accumulation of reactive oxygen species (ROS), and an imbalance of nutrients and ions [2,3]. Food crops such as rice are very sensitive to salt stress [4]. Improving the salt tolerance of rice varieties and expanding their plantation area are important measures to ensure food security [5].

The mechanisms of salt tolerance in rice involve a complex network controlled by multiple genes and strongly influenced by the external environment. Understanding the mechanisms of salt tolerance is very important for breeding salt-tolerant rice varieties [6,7]. The decrease in osmotic pressure causes water loss in plants and a rapid closure of the stomata. One of the main mechanisms of salt tolerance in halophytes is osmotic regulation through the accumulation of osmotic substances (such as sugar, proline, and glycine betaine) in the cytoplasm [8]. Ion imbalance is generally caused by excessive Na^+^ accumulation, and its toxic effects are most often attributed to the inhibition of enzyme activity [9]. Moreover, plants readily take up Na^+^ from the soil. It enters the cells through nonselective cation channels and high-affinity K^+^ ion transporters (HKT) [10]. Plants can maintain a low concentration of Na^+^ by limiting its entry into their cells. Alternatively, Na^+^ can be excluded from the cytoplasm or sequestered into the vacuoles [11]. The major forms of ROS in cells include the superoxide radical (O_2_·^−^), hydrogen peroxide (H_2_O_2_), hydroxyl radical (OH·), singlet oxygen (^1^O_2_), peroxyl radical (ROO·), and alkoxyl radical (RO·). An excessive accumulation of ROS in cells during salt stress may damage lipids, nucleic acids, and proteins, eventually causing plant death [12,13]. At the same time, ROS can be scavenged by enzymatic and nonenzymatic antioxidants. The enzymatic antioxidant system includes superoxide dismutase (SOD), catalase (CAT), ascorbate peroxidase (APX), and peroxidase (POD). Decreased POD activities and increased SOD, APX, and CAT activities can reduce H_2_O_2_ and O_2_^−^ levels and reduce oxidative damage in the radicle and plumule of pak choi [14]. However, some studies have reported that the decreased ROS accumulation in cells with increased POD and CAT activities under salt stress can enhance salt tolerance at the germination stage in rice [15]. Therefore the responses of the antioxidant enzymes vary depending on the time of the treatment and the type of plant tissues and plant species treated [16].

Several genes related to salt tolerance have been reported in rice. Na^+^ can be stored in the vacuoles depending on the upregulation or activity of Na^+^/H^+^ anti-transporters (*AtNHX1*) in the vacuolar membrane [17]. Additionally, the HKT/HAK family genes, including *SKC1*, *OsHKT1;4*, *OsHAK21*, and *ZmHKT1;2*, play an important role in maintaining the balance of K^+^ and Na^+^ in plants [18,19,20,21]. Regulating gene expression to increase a plant’s antioxidation capacity is reported to improve its salt tolerance [22,23,24]. Additionally, some genes related to ROS homeostasis under salt stress have been identified in rice. The overexpression of *OsCu*/*Zn-SOD* could improve rice’s ROS detoxification ability and reduce salt-induced oxidative damage in rice [25]. *OsMADS25* positively regulates the root system and improves salt tolerance in rice via the ABA-mediated regulatory pathway and ROS scavenging [26]. This gene is important for the rice germplasm’s improvement in terms of salt tolerance.

Plant hybrids are extensively used in agriculture because, compared to their parents, they exhibit a better growth rate, biomass accumulation, and resistance to biotic and abiotic stresses [27]. Most of the research on heterosis has focused on the yield of hybrids, and studies on salt stress are scarce. Thus, the effective utilization of stress-resistant hybrids has become an important research hot spot in crop breeding [28,29]. At present, the breeding of salt-tolerant rice varieties is still mainly based on conventional breeding, including the screened salt-tolerant germplasm resources of parents, selecting and breeding salt-tolerant varieties with excellent comprehensive traits through hybridization, and backcrossing to screen salt-tolerant varieties [30,31,32]. Since the 1930s, many salt-tolerant rice varieties, such as Pokkali, Jbona 349, CSR23, Zhongkeyan4, and Nangeng1, have been screened in China and abroad [33,34,35,36,37]. With the development of the third generation of hybrid rice [38], some studies have reported the potential of generating salt-tolerant rice varieties with a high yield by combining *OsRR22* mutants with the “third-generation hybrid rice system” [39]. These varieties have laid a foundation for the popularization and application of rice varieties tolerant to saline–alkali stress.

In this study, we aimed to reveal the possible regulatory mechanisms in rice hybrids and their parents that respond to salt stress. We investigated the phenotypic characteristics, Na^+^ and K^+^ distribution, physiological and biochemical indexes, and alterations in the expression of salt-tolerance-related genes in rice hybrids and their parental lines. The results of this study lay an important foundation for understanding the mechanisms of salt tolerance and further studying the molecular basis of heterosis under salt stress [40].

## 2. Results

### 2.1. Rice Hybrids Exhibited Better Salt Tolerance Than Their Parents

**Figure 1:**
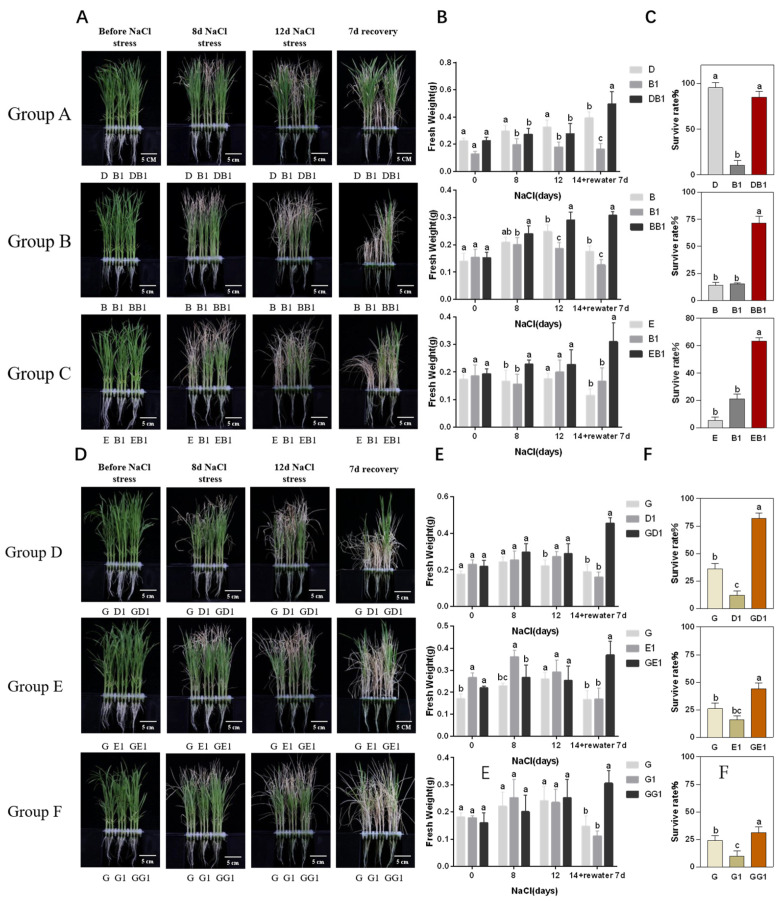
Phenotypes of hybrids and their parents before and under salt stress for 8 or 12 days and on the 7th day of recovery after 14 days of salt stress. (**A**–**C**) Phenotypes, fresh weight, and survival rate of rice hybrids DB1, BB1, and EB1. (**D**–**F**) Phenotypes, fresh weight, and survival rate of rice hybrids GD1, GE1, and GG1. The values shown in (**B**,**E**) are means ± SD (*n* = 5), and those in (**C**,**F**) are means ± SD (*n* = 20–24 plant for each repeat), of three biological replicates, which were statistically analyzed using a one-way ANOVA with the Tukey HSD test. Different lowercase letters indicate that the difference is significant at *p* < 0.05; ns indicate no significant difference.

The phenotypic characteristics of 14 samples (including six rice hybrids and eight parents) (Table 1) were observed on the 0th, 8th, and 12th days after their treatment with 140 mM NaCl. On the 14th day of salt stress, rehydration commenced. The tip of almost all the leaves clearly exhibited symptoms of damage, to varying degrees, on the 8th and 12th days of salt stress (Figure 1A,D). The fresh weight (FW) of the whole plants was measured after different periods of salt stress (Figure 1B,E, Table S1). Differences in the FW were the highest on the 7th day of rehydration after 14 days of salt treatment. The FW of three rice hybrids, Xiang-liang-you-hua-zhan (DB1), Guang-ba-you-hua-zhan (BB1), and Jing-liang-you-hua-zhan (EB1), with hua-zhan (B1) as their parent, did not differ from that of their parental line before salt stress. However, significant differences were observed on the 8th day of salt stress; the FW of BB1 and EB1 was significantly higher, and that of DB1 was lower than that of their parents. However, the FW of DB1 was significantly higher than that of both parents on the 7th day of rehydration. Rice hybrids of Y-liang-you-900 (GD1), Y-liang-you-1 (GE1), and Y-liang-you-2 (GG1) exhibited a FW of more than 0.3 g, with a significantly higher biomass than their parents on the 7th day of rehydration. The survival rate was investigated on the 7th day after their recovery in nutrient solution (Figure 1C,F). The survival rate of hybrid rice was significantly higher than that of the parents, except for in group A. In group A, the survival rate of the rice hybrids (85%) was slightly lower than that of their female parent (95%). In group B, the average survival rates were 14%, 15%, and 71% for B (Guang-ba-A), B1, and BB1, and 5%, 21%, and 63% for Jing-4155s (E), B1, and EB1. Therefore, the average survival rates were significantly higher in rice hybrids. A similar trend was observed in the groups D, E, and G. The lowest survival rate was in the parental line of R900 (D1), R9311 (E1), and Yuan-hui-2 (G1), followed by their respective female parent, Y58s (G). The highest survival rate was observed in the hybrid combinations of GD1, GE1, and GG1. These results indicated that the rice hybrids exhibited superior salt tolerance compared to their parents.

The salt tolerant index (STI) and salinity tolerance level of all varieties were calculated on the 12th day of salt stress (Table 2). Except for group A, almost all rice hybrids exhibited a higher STI than their parents. In group A, the female parent of D exhibited a higher STI value than the rice hybrid DB1. Among the six combinations, BB1 and GD1 exhibited the highest STIs of 83% and 78%, with salt tolerance levels (STL) of 1 and 3, respectively. The hybrid superiority analysis of six hybrid combinations in terms of their STI indicated that the overparent heterosis (OPH) was −18%, 21%, 6%, 26%, 15%, and 11% for DB1, BB1, EB1, GD1, GE1, and GG1, respectively. The hybrid combinations BB1 and GD1 exhibited the highest heterosis. Based on these findings, these two hybrids and their parental lines were selected for a further analysis of their differences in salt tolerance.

### 2.2. Rice Hybrids Exhibited Better Ionic Homeostasis with a Lower Na^+^ Content and Na^+^/K^+^ Ratio

**Figure 2:**
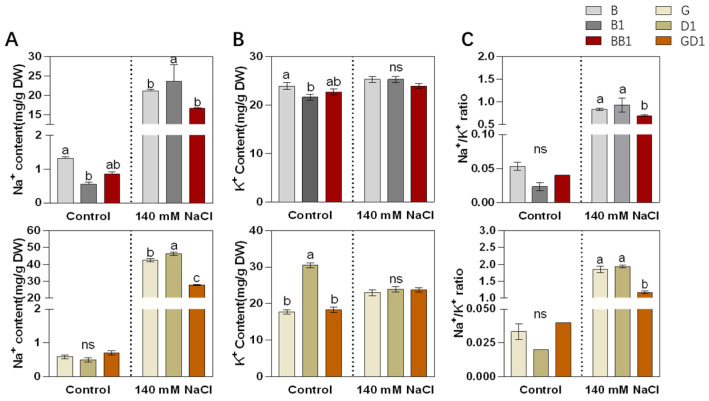
Measurement of the Na^+^ and K^+^ elements in rice shoots that were exposed to salt stress for 10 days. (**A**) The shoots’ Na^+^ contents, (**B**) the shoots’ K+ contents, and (**C**) the shoots’ Na^+^/K^+^ content ratios. The values are means ± SD (*n* = 3), of three biological replicates, which were statistically analyzed using a one-way ANOVA with the Tukey HSD test. Different lowercase letters indicate that the difference is significant at *p* < 0.05; ns indicate no significant difference.

The Na^+^/K^+^ homeostasis in plant shoots is a crucial factor that determines the survival rate of plants under salt stress. The Na^+^ and K^+^ contents in the shoot of the six hybrid samples were measured on the 10th day after treatment with 140 mM NaCl and under the control condition. Further, their Na^+^/K^+^ ratios were calculated. Before the salt treatment, the Na^+^ content of BB1 was approximately 1 mg, which was less than that of B and greater than that of B1. The Na^+^ content of GD1 did not differ from that of G and D1. After salt stress, the Na^+^ contents of B and B1 increased to 21 mg and 23 mg, respectively. However, the Na^+^ content of the rice hybrid BB1 was only 16 mg, which was significantly lower than that of its two parents. A similar trend was observed in GD1 and its parents, with the Na^+^ content of G and D1 increasing to 42 and 46 mg, respectively, while GD1’s was only 27 mg under salt stress, significantly lower than that of its two parents (Figure 2A). The K^+^ contents in all samples under salt stress were not significantly different (Figure 2B). The Na^+^/K^+^ ratios were not significantly different, under normal conditions, between hybrids and parents but they exhibited differences under salt stress, with Na^+^/K^+^ ratios of 8.3%, and 9.3%, and 6.9% for B, B1, and BB1, respectively, and 18.5%, 19.3%, and 11.7% for G, D1, and GD1, respectively. The Na^+^/K^+^ ratios of BB1 and GD1 were much lower than those of their parents (Figure 2C).

### 2.3. Rice Hybrids Exhibited Less ROS Accumulation under Salt Stress

**Figure 3:**
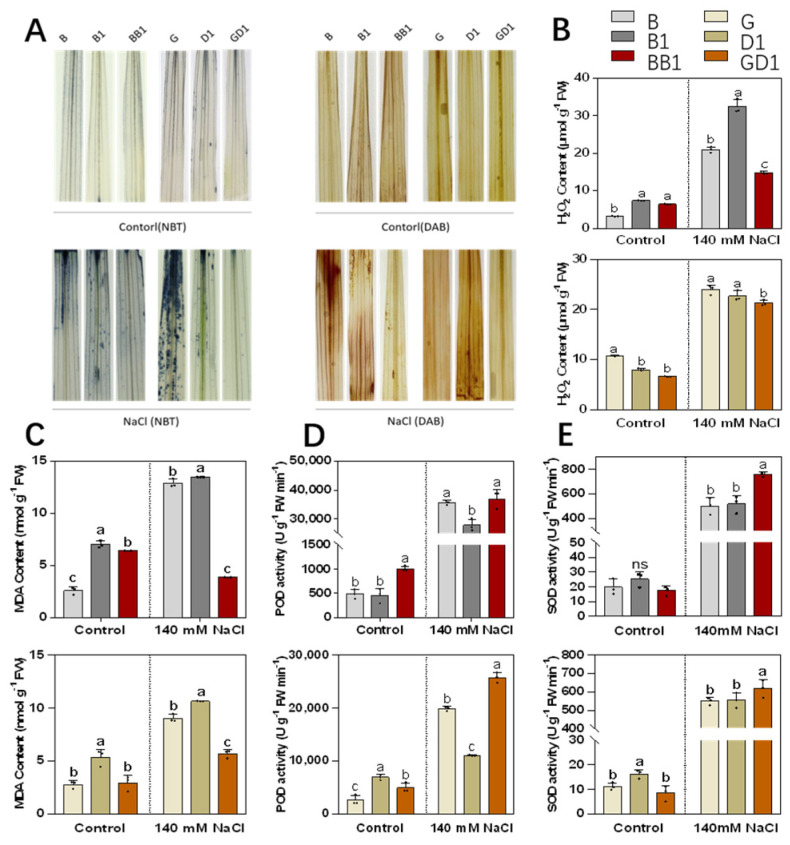
The oxidative stress in the shoots of seedings under salt stress. (**A**) NBT and DAB staining of the leaves of plants exposed to salt stress for 24 h to indicate the O_2_^−^ and H_2_O_2_ in hybrids and their parental lines. (**B**) H_2_O_2_ content (**C**) MDA content, (**D**) POD activity, (**E**) SOD activity. The values shown in (**B**–**E**) are means ± SD (*n* = 3), which were statistically analyzed using a one-way ANOVA with the Tukey HSD test. Different lowercase letters indicate that the difference is significant at *p* < 0.05; ns indicate no significant difference.

Salt stress induces an excess accumulation of ROS and severely damages cellular structures and macromolecules. The contents of O2·^−^ and H_2_O_2_ in the leaves of the six hybrid samples were detected using nitro blue tetrazolium chloride (NBT) and diaminobenzidine (DAB) staining, respectively (Figure 3A). No obvious differences between hybrids and their parents were observed in terms of staining intensity. However, much fewer staining spots were observed in the two hybrids after their exposure to 140 mM NaCl for 24 h, indicating a reduced accumulation of O_2_^−^ and H_2_O_2_ in the hybrids.

Further, the H_2_O_2_ and MDA contents were quantified in the leaves of all six samples exposed to salt stress for 4 days. Under the control condition, the H_2_O_2_ content of BB1 did not vary from that of B1, and it was higher than that of B. However, after salt stress, the H_2_O_2_ content of BB1 was significantly lower than that of its parental line. The H_2_O_2_ content of the rice hybrid GD1 did not differ from that of one of its parents, D1, and it was less than that of its female parent, G, under the control condition. However, under salt stress, the H_2_O_2_ content of GD1 was much lower than that of its parents (Figure 3B). The MDA content of the hybrid BB1 was between that of its parents under the control condition (Figure 3C). However, after salt stress, B and B1 exhibited a significant increase in their MDA content, reaching 12.8 and 13.4 mg, respectively, whereas the hybrid BB1 exhibited a decreasing trend, with an MDA content of 3.8 mg, which was significantly lower than that of its two parents. The MDA contents of GD1 and its parent G (average 2.84 mg) were not significantly different and were lower than that of D1 (5.35 mg), before salt stress. However, after salt stress, the MDA content of the parental lines G and D1 was 9 mg and 10.6 mg, respectively, and that of GD1 was 5.6 mg, which was significantly lower than that of its parents.

The POD enzyme activities of the hybrid rice BB1 was higher than that of its parents both before and after the salt treatment (Figure 3D). The POD enzyme activity of GD1 was between that of G and D1 before the salt treatment but was drastically higher than that of its parents under salt stress (Figure 3D). No difference was observed in terms of SOD enzyme activities between BB1 and its parents before salt stress; however, the hybrid BB1’s SOD enzyme activities were significantly higher than those of its parents after salt stress (Figure 3E). Moreover, the results of the SOD enzyme activity of GD1 did not differ from that of G and were lower than that of D1 before salt stress; however, it was higher than that of its parents under salt stress (Figure 3E). These results suggested that the hybrids were superior in maintaining ROS homeostasis.

### 2.4. Rice Hybrids Exhibited a Higher Expression of Salt-Stress-Responsive Genes

**Figure 4:**
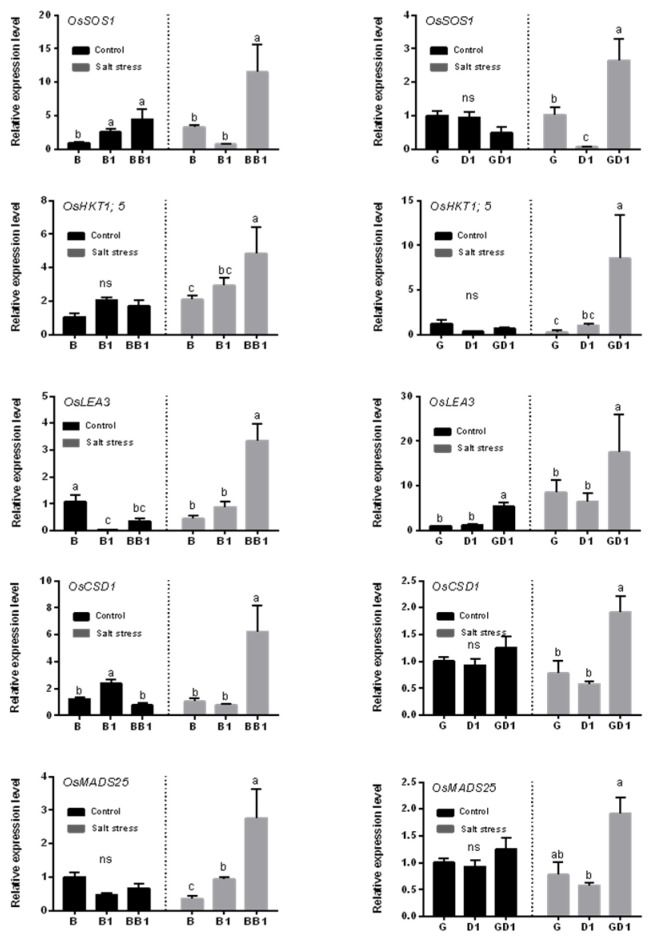
Relative expression levels of the salt-related genes detected using real-time quantitative PCR. The *OsActin* gene was used as the internal control. Data presented are means (±SD), *n* = 3. The different letters represent the significant differences determined using an ANOVA test: *p* < 0.05; ns indicate no significant difference.

The expressions of five salt−stress−responsive genes, including those related to Na^+^ transport (*OsSOS1* and *OsHKT1;5*), antioxidation (*OsCSD1* and *OsMADS25*), and cell structure (*OsLEA3*), were assessed before and after seedlings’ exposure to 140 mM NaCl for 24 h (Figure 4). OsSOS1 is a plasma membrane Na^+^/H^+^ exchange protein that has been reported to be induced by salt stress. Under the control condition, the expression of this gene in BB1 did not vary from that of B1 and was higher than that of B, and no difference was observed in GD1 and its parents, G and D1. After salt stress, this gene was significantly upregulated in the hybrids GD1 and BB1 compared to their respective parents. The expression of *OsHKT1;5* and *OsMADS25* was not significantly different between the hybrids and their parents under the control condition and was significantly increased in the hybrids BB1 and GD1 compared to their parents after the salt stress treatment. The expression of *OsLEA3* in hybrid BB1 was between that of its parental lines before the salt treatment but was significantly enhanced and significantly higher than that of its two parents after salt stress. The expression of *OsLEA3* in the hybrid GD1 was higher than that of its two parents under both the control and salt stress conditions. Compared to the control condition, *OsCSD1* genes was relatively higher in hybrids BB1 and GD1, and relatively lower in their respective parents (B, B1, G, and D1) after salt stress. In summary, after the salt treatment, the expression of all five genes increased in the hybrids. These results indicate that the hybrids BB1 and GD1 may exhibit salt tolerance via regulating their expression of salt-stress-related genes.

## 3. Discussion

Rice is the first choice for the improvement of coastal beaches and saline–alkali land because of its salt tolerance. In the past few decades, a number of salt-tolerant rice varieties and germplasms, such as Pokkali, Nona Bokra, and SeaRice 86, have been discovered or cultivated [41,42,43]. Utilizing the hybrid advantages of rice to breed hybrids with outstanding salinity tolerance compared to conventional varieties is an important strategy for the future. Plant hybrids are extensively used in agriculture due to their improved growth rate, biomass accumulation, and resistance to various biotic and abiotic stresses compared to their parents. Thus, the utilization of heterosis to increase rice biomass and yield would be a preferred way to meet the demand for rice cultivation in saline–alkali soils. Our results demonstrated the better salt-tolerance performance of two rice hybrids and elucidated the complex regulatory mechanisms in rice, involving multiple genes and pathways, that allow it to adapt to salt stress.

In this study, we analyzed the salt tolerance phenotypes (FW and survival rate) of six groups, including eight parents and three hybrid combinations. Further, their STI and STL were calculated based on their DW, and heterosis analysis was performed based on their STI. On the 7th day of rehydration after 14 days of salt stress, all the rice hybrids exhibited a significant increase in their FW compared to their parents. A higher biomass may enhance salt tolerance. The survival rate of the hybrids BB1, EB1, GD1, GE1, and GG1 was significantly higher than that of both their parents after 7th days of rehydration, whereas that of DB1 and its parent D did not differ. The STI analysis of group A revealed that the female line D had the same STL as its hybrid but had higher STI than the hybrid DB1. This is consistent with previous studies [44], suggesting that D has an advantageous biomass for NaCl treatment. The other five rice hybrids had higher STIs than their parents. Among the six hybrid combinations, BB1 and GD1 exhibited the highest STIs, with strong salt tolerance. Further, the superiority of the hybrids was analyzed based on their STIs. The hybrids BB1 and GD1 exhibited OPH of 21% and 26%, respectively, with strong heterosis. Furthermore, two hybrids (BB1 and GD1) exhibiting higher STIs and heterosis were selected for further study.

Maintaining a low Na^+^/K^+^ ratio and reducing the Na^+^ accumulation in plants are essential for salt tolerance because they reduce ion toxicity and contribute to the recovery of metabolic processes [45]. It has been reported that the increased K^+^ uptake under high Na^+^ concentrations can effectively improve Na^+^/K^+^ homeostasis under salt stress [46]. Meanwhile, some studies have reported that plants could reduce Na^+^ accumulation through increasing their Na^+^ exclusion under salt stress [47,48]. In our study, the hybrids BB1 and GD1 exhibited lower Na^+^ contents and Na^+^/K^+^ ratios than their parental line. Our results suggested that, under salt stress, hybrids may reduce ion toxicity and increase their salt tolerance by absorbing, excluding, or segregating Na^+^.

In the case of physiological traits, salt stress has been proven to cause an excessive accumulation of ROS, which damages cells and tissues. Maintaining the dynamic balance of ROS homeostasis is one of the important mechanisms in enhancing the tolerance of plants to abiotic stress [49]. Higher activities of antioxidative enzymes can improve plants’ ROS scavenging ability, which helps to increase their salt tolerance. Our results revealed that the H_2_O_2_ and MDA contents were significantly lower and the POD and SOD activities were significantly higher in hybrid rice seedlings than in their parents after salt stress. Moreover, the DAB and NBT staining intensities were significantly lower in the hybrids than in their parents. These results indicated that scavenging ROS and controlling membrane damage are important during salt stress.

All genes in hybrids are derived from both their parents, and no new genes emerge. However, the gene expression levels vary between hybrids and their parents, resulting in phenotypic differences [50,51]. The Na^+^/H^+^ anti-transporter (OsSOS1) in the plasma membrane of rice promotes Na^+^ efflux to the apoplast, reducing the net cellular uptake of Na^+^ and maintaining a low Na^+^/K^+^ ratio in the cells [52,53]. The OsHKT1;5 transporter protein plays a role in reducing Na^+^ accumulation by returning excess aboveground Na^+^ to the roots through xylem unloading, leading to less Na^+^ toxicity in the leaves during salt stress [54]. OsLEA3 is a late embryogenesis abundant group 3 protein. In previous studies, the protein level of OsLEA3 was significantly different between salt-tolerant and salt-sensitive rice materials [55]. Our results revealed that the higher expression of *OsSOS1* and *OsHKT1;5* under salt stress resulted in a lower Na^+^/K^+^ ratio. Meanwhile, the increased levels of *OsLEA3, OsCSD1,* and *OsMADS25* expression in hybrids could significantly decrease the membrane damage caused by salt stress, conferring salt tolerance to rice.

## 4. Materials and Methods

### 4.1. Plant Material and Growth Conditions

In total, 14 rice cultivars were provided by the Hunan Hybrid Rice Research Centre, which included 6 hybrids and 8 parental lines. All seeds were hydrated in distilled water at 37 °C for 48 h. After germination, all plants were grown in a greenhouse at 26 ± 2 °C, a humidity of 60%, and a light/dark cycle of 14 h/10 h. The rice seedlings were cultured in Yoshida nutrient solution salts [56] for 14 days, and the medium was changed every 3 days. Further, they were treated with or without 140 mM NaCl. All the above treatments were performed with three biological replicates.

### 4.2. Phenotype Analysis and Evaluation of Salinity Tolerance

To observe the salt tolerance phenotypes of all samples, photographs were taken on the 0th, 8th, and 12th days after salt treatment and on the 7th day of rehydration after the 14-day salt treatment. Yoshida nutrient solution was used for rehydration. At the same time, samples were randomly selected from each five plants for FW measurements. After rehydration, their respective survival rates were assessed by observing the state of the leaves; plants with yellowish brown and wilted leaves were considered dead plants, and those with green and upright leaves were considered alive plants. Rice seedlings from the 12th day with and without salt treatment were stored at 80 °C for 3 days and were used to measure DW. Each treatment consisted of five biological replicates. The STI was calculated based on the sample’s relative DW under salt stress (Table S2) [57]. The formula used is as follows:

$$salt\;tolerant\;index(STI;\;\%)=\frac{DW\;of\;the\;salt\;stress\;treatment}{DW\;of\;the\;control\;treatment}\times 100$$

The breeding lines with 0–20%, 20–40%, 40–60%, 60–80%, and 80–100% STIs were classified as having extremely weak salt tolerance (level 9), weak salt tolerance (level 7), medium salt tolerance (level 5), strong salt tolerance (level 3), and extremely strong salt tolerance (level 1), respectively. The values of OPH were determined based on the STI using the following formula:

OPH = (F1 − Ph)/Ph × 100%

where F1 is the performance of the first generation (hybrid) and Ph is the performance of the best parent. Images of the phenotypes were captured on the specified dates, and representative data were provided.

### 4.3. Measurement of Na^+^ and K^+^ Contents

On the 10th day of the salt stress treatment, 30 seedlings treated with or without 140 mM NaCl were collected. Their shoots were washed with distilled water and dried at 80 °C for 3 days. Further, 0.2 g of this dried sample was digested with 6 mL HNO_3_ for 2 h and further diluted with 2 mL of deionized water, followed by heating at 180 °C for 15 min (MARS XPRESS, CEM Corporation, Matthews, NC, USA). The Na^+^ and K^+^ contents were measured using inductively coupled plasma-mass spectrometry (ICP-MS) (Thermofisher Scientific iCAP RQ, Waltham, MA, USA), and the Na^+^/K^+^ ratio was calculated (Table S3).

### 4.4. Measurement of Antioxidant Enzyme Activity

After 4 days of salt treatment, 0.1 g leaf samples were collected and placed in liquid nitrogen. We added 1 mL (pH 7.8) of phosphoric acid buffer and centrifuged at 8000× *g* for 20 min at 4 °C. The SOD and POD activities were measured according to the protocol established by Spitz DR and Reuveni R [58,59]. The SOD and POD activities of samples were analyzed by measuring their absorbance at 560 and 470 nm, respectively, using a spectrometer (BioTec Synergy LX, Synergy Corporation, Houston, TX, USA).

### 4.5. Measurement of the Total H_2_O_2_ and MDA Contents

The H_2_O_2_ content of samples was determined using a Hydrogen Peroxide Assay Kit (Solaibao Technology, Beijing, China) according to the manufacturer’s instructions. In total, 0.1 g leaf samples with or without the 140 mM NaCl treatment were mixed with 1 mL of propanone and centrifuged at 8000× *g* for 10 min at 4 °C. Further, 250 μL of supernatant and 325 μL of test solution (5% titanic sulfate) were mixed and placed at 25 °C for 10 min. The absorbance was immediately measured at 415 nm using a spectrometer. The H_2_O_2_ content in the sample was calculated using its calibration curve. The MDA content was determined using a Malondialdehyde (MDA) content Assay Kit from Solarbio Technology (Beijing, China). Rice leaves were homogenized in 1 mL of 10% trichloroacetic acid (TCA) and centrifuged at 8000× *g* for 10 min at 4 °C. Further, 100 μL of the supernatant was mixed with 200 μL of 0.67% thiobarbituric acid (TBA) and placed in a boiling water bath for 60 min. Further, the mixture was centrifuged at 4000× *g* and 4 °C for 10 min, and the absorbance of the supernatant was measured at 450, 532, and 600 nm.

### 4.6. Histochemical Staining

NBT and DAB staining was utilized to detect the cellular accumulation of superoxide (O_2_^−^) and hydrogen peroxide (H_2_O_2_). Briefly, isolated leaves were immersed in NBT and DAB staining solution (Coolaber Technology, Beijing, China), vacuumed for 40 min, incubated overnight at room temperature, soaked in 95% ethanol, and placed in a water bath at 80 °C until decolorized. Then, they were observed under a stereo microscope.

### 4.7. Total RNA Extraction and Gene Expression Analysis

Total RNA was extracted from the leaves of seedlings subjected to 12 h of salt stress or control conditions using total RNA extraction kits (TransGen Biotech, Beijing, China). cDNA was synthesized from 1 µg of total RNA using a PrimeScript™ II 1st Strand cDNA Synthesis Kit (Takara Biomedical Technology, Beijing, China). A quantitative real-time PCR (qRT-PCR) was conducted using a LightCycler 480 System (Roche Diagnostics, Rotkreuz, Switzerland) and TB Green Premix Ex Taq (Takara Biomedical Technology). The relative expression levels of the selected genes were calculated using the 2^−∆∆CT^ method [60]. *OsActin* was used as the internal reference. The genes and primers used for the qRT-PCR are given in Table S4. The experiment was performed in triplicate for each sample.

### 4.8. Statistical Analysis

All experiments were performed at least three independent times. Data were analyzed using an ANOVA and GraphPad Prism version 7.0 for Windows (GraphPad version 7.0 Software, Boston, MA, USA, accessed on 1 January 2022, www.graphpad.com). *p* < 0.05 (Tukey’s test) was considered significant.

## 5. Conclusions

In conclusion, this study has provided valuable insights into the mechanisms of rice hybrids’ tolerance to salt stress. However, additional research is needed to further elucidate these mechanisms and develop more effective strategies for rice cultivation in saline–alkali soils.

## Figures and Tables

**Table 1:** Information about the cultivars used in the experiment.

Group	Cultivars	Letter
A	Guang-Xiang-24 (♀)	D
	Hua-Zhan (♂)	B1
	Xiang-Liang-You-Hua-Zhan (F1)	DB1
B	Guang-Ba-A (♀)	B
	Hua-Zhan (♂)	B1
	Guang-Ba-You-Hua-Zhan (F1)	BB1
C	Jing-4155S (♀)	E
	Hua-Zhan (♂)	B1
	Jing-Liang-You-Hua-Zhan (F1)	EB1
D	Y58S (♀)	G
	R900 (♂)	D1
	Y-Liang-You-900 (F1)	GD1
E	Y58S (♀)	G
	R9311 (♂)	E1
	Y-Liang-You-1 (F1)	GE1
F	Y58S (♀)	G
	Yuan-Hui-2 (♂)	G1
	Y-Liang-You-2 (F1)	GG1

**Table 2:** Analysis results of heterosis differences and STIs of different hybrid combinations (%).

Groups	Cultivars	Dry Weight	Salt Tolerant Index (%)	Salt Tolerance Level	Overparent Heterosis (%)
Group A	CK-D	0.07 ± 0.01	79	3	−18
	Salt-D	0.06 ± 0.01			
	CK-B1	0.08 ± 0.02	52	5	
	Salt-B1	0.04 ± 0.01			
	CK-DB1	0.11 ± 0.01	64	3	
	Salt-DB1	0.07 ± 0.01			
Group B	CK-B	0.07 ± 0.02	68	3	21
	Salt-B	0.05 ± 0.01			
	CK-B1	0.08 ± 0.01	59	5	
	Salt-B1	0.04 ± 0.01			
	CK-BB1	0.02 ± 0.03	83	1	
	Salt-BB1	0.07 ± 0.01			
Group C	CK-E	0.13 ± 0.02	40	7	6
	Salt-E	0.05 ± 0.01			
	CK-B1	0.07 ± 0.01	48	5	
	Salt-B1	0.03 ± 0.00			
	CK-EB1	0.09 ± 0.01	51	5	
	Salt-EB1	0.04 ± 0.01			
Group D	CK-G	0.10 ± 0.01	59	5	26
	Salt-G	0.06 ± 0.01			
	CK-D1	0.10 ± 0.01	62	3	
	Salt-D1	0.06 ± 0.01			
	CK-GD1	0.07 ± 0.01	78	3	
	Salt-GD1	0.05 ± 0.01			
Group E	CK-G	0.12 ± 0.01	57	5	15
	Salt-G	0.06 ± 0.01			
	CK-E1	0.11 ± 0.03	34	7	
	Salt-E1	0.04 ± 0.01			
	CK-GE1	0.05 ± 0.01	66	3	
	Salt-GE1	0.04 ± 0.01			
Group F	CK-G	0.10 ± 0.03	48	5	11
	Salt-G	0.05 ± 0.01			
	CK-G1	0.08 ± 0.01	55	5	
	Salt-G1	0.04 ± 0.01			
	CK-GG1	0.07 ± 0.01	62	3	
	Salt-GG1	0.04 ± 0.01			

## Data Availability

The data presented in this study are available within the article.

## Supplementary Materials

The following supporting information can be downloaded at https://www.mdpi.com/article/10.3390/plants13050744/s1, Table S1: statistics on fresh weight; Table S2: statistics on the Salt Tolerant Index; Table S3: statistics on Na+ and K+ content; Table S4: the primer sequences used in this experiment.
